# Regulation of Apoptosis by the Bcl-2 Family of Proteins: Field on a Brink

**DOI:** 10.3390/cells9092121

**Published:** 2020-09-18

**Authors:** Alexey S. Ladokhin

**Affiliations:** Department of Biochemistry and Molecular Biology, University of Kansas Medical Center, Kansas City, KS 66160, USA; aladokhin@kumc.edu; Tel.: +913-588-0489; Fax: +913-588-7440

**Keywords:** apoptosis, mitochondrial outer membrane permeabilization, protein–membrane interactions, Bcl-2 proteins as therapeutic targets, cancer

Apoptosis, a form of programmed cell death, is a highly regulated process critical for tissue development, homeostasis, and pathogenesis of various diseases. Hyperactive apoptosis, for example, contributes to neurodegeneration and immunodeficiency, while insufficient apoptosis leads to autoimmunity and cancer. Furthermore, the ability of cancer cells to avoid apoptosis significantly complicates treatment. The critical step in triggering apoptosis is the permeabilization of the mitochondrial outer membrane (MOMP), which releases apoptotic factors into the cytosol and leads to cell death. MOMP is controlled and executed by the numerous proteins of the Bcl-2 family. The current Special Issue of *Cells*, “Regulation of Apoptosis by the Bcl-2 Family of Proteins”, gives a sense of the recent advances in all aspects of apoptotic regulation, ranging from in vitro biophysical characterization, through in vivo cellular studies, to preclinical and clinical studies of cancer treatments. The contributions to this Special Issue include three original articles, employing sophisticated techniques to gain important insights into the mechanisms of apoptotic regulation [1,2,3]; a thought-provoking perspective article [4] and two conceptual reviews, discussing state-of-the-art developments in drugs targeting anti-apoptotic Bcl-2 proteins [5,6]. Despite the collection of articles not encompassing all aspects of apoptosis, the following two general themes can be clearly identified.

*The Curious Puzzle of Membrane-Controlled Regulation of Bcl-2 Proteins*. In spite of the recent advances in solving the structures of the *soluble* conformations of most of Bcl-2 proteins, the exact mechanism of their actions remains unresolved. This is primarily because the functionally-important conformations are induced by interactions with the *membrane*. A major knowledge gap is, therefore, the lack of accurate molecular pictures of protein-lipid interactions, protein refolding on the membrane and protein–protein interactions in the context of membrane-refolded conformations. The perspective article by Flores-Romero and García-Sáez [4], entitled *The Incomplete Puzzle of the BCL2 Proteins*, provides a critical examination of the recent insights and remaining challenges in this excitingly complex field of Bcl-2 proteins. It concludes that “we still fail to understand the contribution of mitochondrial lipids in modulating their activation, oligomerization and formation of supramolecular structures at apoptotic foci during and after MOMP.” One step toward completing the outlined puzzle is presented by Vasquez-Montes and co-workers [3], who use a battery of spectroscopic tools including single-molecule Förster Resonance Energy Transfer to test the hypothesis of the lipid-dependent conformational switching in the apoptotic inhibitor Bcl-xL. Specifically, they demonstrate that the membrane insertion of Bcl-xL results in the release of the BH4 domain from the folded structure, thus switching from canonical (BH3/grove-dependent) to non-canonical (BH4-dependent) modes of apoptotic inhibition.

*Apoptotic Restoration as Anti-Cancer Strategy.* Inhibiting the inhibitor has been the main approach in the development of cancer therapeutics targeting the Bcl-2 family. Two reviews describe current progress with applications of BH3 mimetic inhibitors, such as a drug recently approved by the FDA, venetoclax (ABT-199), which restores apoptosis and primes malignant cells for death [5,6]. Another important aspect of the functioning of the Bcl-2 proteins is the involvement of family members in various cellular processes and interactions, not directly related to MOMP. For example, Han and co-workers [2] present evidence of Bcl-xL being involved in the regulation of autophagy, while Kuo and co-workers [1] demonstrate that inhibiting deubiquitinating enzymes enhances the cisplatin-induced antitumor effect via concurrent suppression of Bcl-2 protein levels.

As illustrated by the contributions to this Special Issue, the field of apoptotic regulation by Bcl-2 proteins is at a peculiar place, where advancements in targeted therapies coexist with a lack of mechanistic insights into MOMP regulation. It looks, though, like both the biomedical and biophysical/cellular branches of studies are beginning to converge to produce a comprehensive picture of the fascinating process of apoptotic regulation, allowing for the acceleration of knowledge-based drug design. 
